# Protective effects of chitosan based salicylic acid nanocomposite (CS-SA NCs) in grape (*Vitis vinifera* cv. ‘Sultana’) under salinity stress

**DOI:** 10.1038/s41598-023-27618-z

**Published:** 2023-01-17

**Authors:** Mohammad Ali Aazami, Maryam Maleki, Farzad Rasouli, Gholamreza Gohari

**Affiliations:** Department of Horticulture, Faculty of Agriculture, University of Maragheh, Maragheh, Iran

**Keywords:** Physiology, Plant sciences

## Abstract

Salinity is one of the most important abiotic stresses that reduce plant growth and performance by changing physiological and biochemical processes. In addition to improving the crop, using nanomaterials in agriculture can reduce the harmful effects of environmental stresses, particularly salinity. A factorial experiment was conducted in the form of a completely randomized design with two factors including salt stress at three levels (0, 50, and 100 mM NaCl) and chitosan-salicylic acid nanocomposite at three levels (0, 0.1, and 0.5 mM). The results showed reductions in chlorophylls (a, b, and total), carotenoids, and nutrient elements (excluding sodium) while proline, hydrogen peroxide, malondialdehyde, total soluble protein, soluble carbohydrate, total antioxidant, and antioxidant enzymes activity increased with treatment chitosan-salicylic acid nanocomposite (CS-SA NCs) under different level NaCl. Salinity stress reduced Fm', Fm, and Fv/Fm by damage to photosynthetic systems, but treatment with CS-SA NCs improved these indices during salinity stress. In stress-free conditions, applying the CS-SA NCs improved the grapes' physiological, biochemical, and nutrient elemental balance traits. CS-SA NCs at 0.5 mM had a better effect on the studied traits of grapes under salinity stress. The CS-SA nanoparticle is a biostimulant that can be effectively used to improve the grape plant yield under salinity stress.

## Introduction

Salinity stress is one of the most important environmental stresses that threaten agricultural production worldwide^1^. Salinity hinders growth, photosynthesis, transpiration, and stomata conductance. It increases the reactive oxygen species (ROS) content in plant cells, resulting in ion poisoning and impaired ion homeostasis. Therefore, it causes an imbalance in nutrient uptake and destruction of various membranes leading to osmotic and ionic stress^2^. The absorption of toxic sodium and chlorine ions restricts nutrient uptake, transport, and distribution, resulting in a nutritional imbalance in the plant^3–5^. Salinity stress causes an ionic imbalance in the cell due to the overaccumulation of Na^+^ and Cl^−^, which reduces the uptake of nutrients such as nitrogen, potassium, calcium, magnesium, and manganese, thereby inhibiting plant growth^6^. The accumulation of Na^+^ and Cl^−^ in leaves reduces the leaf photosynthetic area in plants, ultimately affecting plant performance^7^. ROS accumulation under salinity stress causes damage to photosynthetic pigments and chlorophyll degradation^8,9^. Many plants commonly react to salinity through the overproduction of a set of organic compounds to ameliorate its harmful effects. These compounds are carbohydrates, amino acids, and proteins that act as osmolytes to counteract stress^10^. In addition, plants containing dynamic antioxidant enzymes can reduce damage from environmental stressors. An efficient antioxidant system shows good tolerance to environmental stress and salinity^11,12^.

The effectiveness of salicylic acid (SA) in resistance to environmental stresses in plants has been documented in numerous studies. The effective application of SA in salinity stress of grapes cv. ‘Sultana’ led to significantly increased salinity tolerance by reducing the Na^+^/K^+^ ratio, leaf electrolyte leakage, MDA, and H_2_O_2_ and increasing proline and enzymatic activities (POD, APX, CAT, and SOD)^13,14^.

Chitosan acts as a biostimulant and a potential stimulant in agriculture. This non-toxic, biodegradable, and biocompatible substance reduces the adverse effects of abiotic stresses through the stress transfer pathway by secondary signaling^15^. In addition, chitosan upgrades several defensive genes in plants such as pathogenesis-related genes (glucanase and chitinase)^16^. Chitosan reduces the effect of salinity stress on plants and enhances plant growth by regulating cellular osmotic pressure by increasing the availability and uptake of water and essential nutrients^17^. Chitosan biopolymer has been used in plant growth and protection, particularly as a nano-coating for chemicals^18,19^. Treatment with chitosan improved the antioxidant potential in different tissues of *V. vinifera*. Antioxidant activity and antimicrobial compounds increased in different components of grapes treated with chitosan solution^20^. SA-functionalized chitosan nanoparticles have greater potential in improving plant immunity as they are involved in the transfer of plant signals^19^. CS-SA NCs were studied as a biostimulant to enhance plant defense and growth. The results showed that CS-SA NCs expressed considerable physiological-biochemical reactions in vitro and in vivo. These responses occur as high activities of antioxidant defense enzyme, modulation of ROS, reinforcement of cell wall by lignin deposition, disease control, and plant growth^21^. Considering that SA and CS have shown effective effects on plant growth, physiological, and biochemical parameters, especially anthocyanins and enzymes, the combination of nanoparticles (CS-SA NCs) may have a synergistic effect due to the effect of chitosan^22^ CS-SA treatments with the lowest encapsulated ratio reduced the toxicity of free SA on Arabidopsis thaliana. In this system, plants treated with capsule, CS-SA had more root and rosette growth than plants treated with free SA^23^.

Grape (*Vitis vinifera* L.) is a perennial plant belonging to the Vitaceae family that is widely cultivated around the world and is one of the most economically important fruit crops^24^. Grape growth and development are affected by abiotic stresses such as drought, salinity, extreme temperatures, chemical toxicity, and oxidative stress^25^. Salinity reduces grape biomass production and, ultimately, the death of the whole plant. Leaf, root, and shoot biomasses decreased in the grape plant under salinity stress^26^. A positive correlation between osmolytes accumulation and stress tolerance has been reported in several studies^27^. Fozouni et al.^28^ showed that in the response of four cultivars of rooted table grapes (*Vitis vinifera* L.) to different concentrations of salt, proline accumulation increased significantly with increasing salinity.

Based on previous research on the beneficial effects of SA and chitosan on abiotic stresses and the other hand the positive effect of nanocomposites, the response of grapes cv. ‘Sultana’ to salinity stress and the role of a CS-SA nanocomposite in reducing the effects of salt were studied through the antioxidant defense system, fluorescence chlorophyll, and ionic homeostasis.

## Results

### Chlorophyll fluorescence parameters

According to the results, chlorophyll fluorescence parameters were significantly affected by salinity stress and foliar application of CS-SA NCs at 1% and 5% probability (Table 1). The salinity stress and foliar application along with the CS-SA NCs on the photosynthetically active radiation (PAR) value revealed that the foliar application in 0 and 50 mM salinity treatments did not significantly affect the PAR value. The highest PAR values were observed in 0.5 mM of CS-SA NCs under 100 mM salinity. Our findings indicated that salinity stress and the foliar application of the nanocomposite did not significantly influence the minimum fluorescence value (F_0_). But, maximum fluorescence (Fm) decreased with increasing salinity stress and the mentioned treatments, so the highest Fm value was observed in the treatment with 0.5 mM of CS-SA NCs without salinity stress. Also, the application of CS-SA NCs (0.5 mM) at different salinity levels increased the electron transfer rate (ETR). Fm' decreased with enhancing salinity stress, but this reduction was significantly higher in unsprayed samples than in the plants treated by CS-SA NCs. Based on the obtained results, the highest maximum quantum efficiency of photosystem II (Fv/Fm) was observed in 0.5 mM of CS-SA NCs without salinity, and the lowest belonged to 100 mM salinity treatment and no foliar application (Table 1).

**Table 1:** Mean comparisons for the effects of CS-SA NCs under salinity on the Chlorophyll fluorescence of grapevine cv. ‘Sultana’.

NaCl (mM)	CS-SA NCs (mM)	PAR	Fm'	ETR	Fm	Fv/Fm
	0	11 ± 1.41c	2.31 ± 0.09d	2.65 ± 0.35cd	3.01 ± 0.09ab	0.56 ± 0.02c
0	0.1	9.5 ± 2.56cd	2.79 ± 0.04cd	2.66 ± 0.68cd	2.79 ± 0.04bc	0.67 ± 0.01ab
	0.5	13.5 ± 3.06bc	3.57 ± 0.23a	3.85 ± 0.92bc	3.57 ± 0.23a	0.79 ± 0.08a
	0	19.5 ± 0.23ab	2.36 ± 0.23d	4.8 ± 0.37ab	2.36 ± 0.23c	0.6 ± 0.03b
50	0.1	16 ± 1.41abc	2.38 ± 0.50d	4.1 ± 0.33bc	2.38 ± 0.05c	0.61 ± 0.04b
	0.5	19.33 ± 0.27ab	2.70 ± 0.11cd	5.35 ± 0.02ab	2.7 ± 0.11bc	0.67 ± 0.03ab
	0	11.5 ± 3.53c	2.66 ± 0.02cd	3.4 ± 1.03bc	1.31 ± 0.02d	0.45 ± 0.01d
100	0.1	14.06 ± 0.13bc	3.11 ± 0.06ab	2.63 ± 0.14cd	2.36 ± 0.06c	0.65 ± 0.02ab
	0.5	22.66 ± 0.72a	3.20 ± 0.02ab	6.3 ± 0.17a	2.78 ± 0.02bc	0.53 ± 0.03c
Significance						
Salinity		**	**	*	**	**
Nanocomposite (NCs)		**	**	**	**	**
Salinity × NCs		*	**	*	**	**
Error		16.59	0.14	1.45	0.155	0.003
CV		23.94	13.53	21.39	14.43	8.37

Similar letters show no meaningful difference at 5% probability level by Duncan’s Multiple Range Test. Data are mean ± SD (n = 3 replicates). ns, ** and *: Non-significant, significant at 1 and 5 percentage probability levels, respectively.

### Chlorophyll and carotenoid content

**Figure 1:**
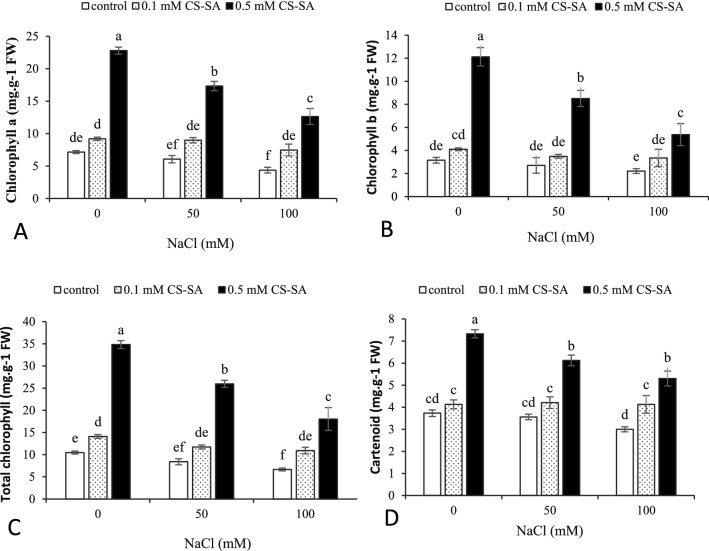
Effect of CS-SA NCs on Chlorophyll a (**A**), b (**B**), total chlorophyll (**C**) and carotenoid (**D**) of grapevine cv. ‘Sultana’ under salinity stress. Means followed by the same letter on columns are not significantly different at 0.05 level, according to Duncan's multiple range test. Data are mean ± SD (n = 3 replicates).

The results showed that chlorophyll a, b, and total, and carotenoids content were significantly influenced by salinity stress and foliar application of CS-SA NCs at 1% and 5% probability (Table 2). Based on the results of the comparison of means, the highest chlorophyll a content (22.8 mg/g FW) was measured in without salinity along with 0.5 mM of CS-SA NCs, and the lowest was recorded in grape plants under 100 mM salinity and without any foliar application. also chlorophyll b content reduced using salinity stress that the maximum obtainedat 0.5 mM of CS-SA NCs in stress-free conditions, and the lowest content was observed in 100 mM salinity treatment and no CS-SA NCs foliar application. The application of CS-SA NCs (0.5 mM) at different salinity levels significantly increased the total chlorophyll compared to the other treatments. The foliar application significantly increased leaf carotenoid content and the highest was obtained from the plants sprayed with 0.5 mM of CS-SA NCs and stress-free conditions (Fig. 1).

**Table 2:** Analysis of variance chlorophylls and carotenoid of grapevine cv. ‘Sultana’ treated with CS-SA NCs under salinity stress.

S.O.V	df	Chlorophyll a	Chlorophyll b	Total chlorophyll	Carotenoid
Salinity	2	54.061**	17.93**	142.107**	1.918**
Nanocomposite (NCs)	2	339.907**	92.821**	788.458**	19.2**
Salinity × NCs	4	16.048**	8.75**	44.479**	0.837*
Error	18	1.958	0.887	4.51	0.236
CV		13.11	18.81	13.45	10.51

*,** significant at *p* ≤ 0.05 and *p* ≤ 0.01 respectively.

### Osmolytes and membrane stability

**Figure 2:**
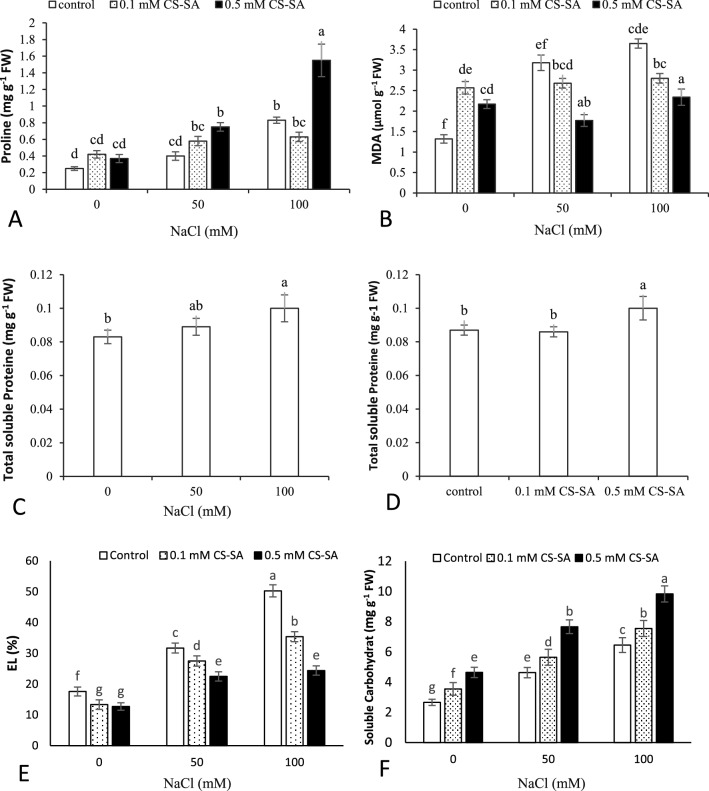
Effect of CS-SA NCs on Proline (**A**), MDA (**B**), Total soluble protein (**C**, **D**), EL (**E**) and Soluble carbohydrate (**F**) of grapevine cv. ‘Sultana’. Means followed by the same letter on columns are not significantly different at 0.05 level, according to Duncan's multiple range test. Data are mean ± SD (n = 3 replicates).

Based to the ANOVA, salinity stress and foliar application of CS-SA NCs had a significant impact on proline, MDA, carbohydrate, and electrolytes at 1% probability, while the effects were not significant on protein content (Table 3). The results showed that leaf proline content enhanced with increasing salinity stress. The 100 mM salinity and 0.5 mM of CS-SA NCs foliar application contained the highest proline content, and the lowest was observed in the control plants. Lipid peroxidation of the membrane boosted under salinity stress. The highest content of MDA belonged to the grape plants supplemented with 100 mM salinity and no foliar application treatments, and the lowest amount was found in the control. Increasing the concentration of CS-SA NCs foliar treatment improved the total soluble protein content. Electrolyte leakage increased with increasing salinity stress. Application of 0.5 mM CS-SA NCs caused a significant reduction in electrolyte leakage at 100 mM NaCl salt stress. Application of CS-SA NCs under salt stress increased the soluble carbohydrates in grape plants (Fig. 2).

**Table 3:** Analysis of variance osmolites and electrolyte leakage and total antioxidant of grapevine cv. ‘Sultana’ treated with CS-SA NCs under salinity stress.

S.O.V	df	Proline	MDA	Protein	Carbohydrate	Electrolyte leakage
Salinity	2	1.007**	1.888**	0.001**	42.303**	1111.577**
Nanocomposite (NCs)	2	0.423**	3.952**	0.001**	18.122**	322.522**
Salinity × NCs	4	0.197**	0.06**	0.0001^ns^	0.471**	134.147**
Error	18	0.025	0.122	0.0001	0.039	1.634
CV (%)		24.32	13.95	10.3	3.39	4.89

*,**, ns significant at *p* ≤ 0.05, *p* ≤ 0.01 respectively and no significant.

### Biochemical parameters

**Figure 3:**
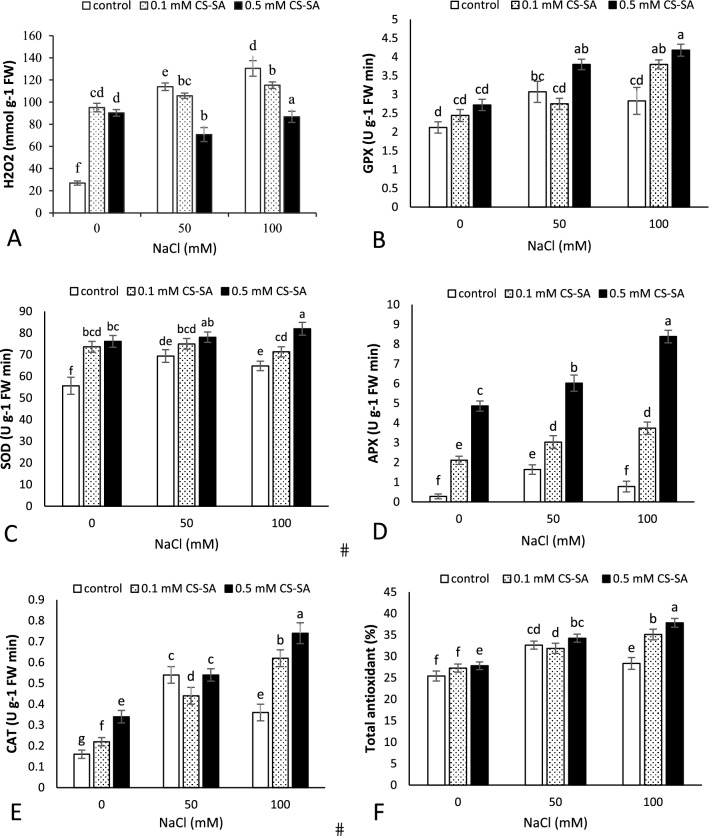
Effect of CS-SA NCs on H_2_O_2_ (**A**), GPX (**B**), SOD (**C**), APX (**D**), CAT (**E**) and total antioxidant (**F**) of grapevine cv. ‘Sultana’. Means followed by the same letter on columns are not significantly different at 0.05 level, according to Duncan's multiple range test. Data are mean ± SD (n = 3 replicates).

According to the findings, H2O2, enzyme antioxidant activity, and total antioxidant activity were significantly affected by salinity stress and foliar application of CS-SA NCs at 1% and 5% probability (Table 4). H_2_O_2_ levels were maximized at 100 mM salinity level without foliar application, and the lowest belonged to the control treatment. The GPX activity increased in the plants subjected to salinity stress, and the uppermost was observed in 100 mM salinity stress along with 0.5 mM of CS-SA NCs. The increasing salinity level led to a significant enhancement in SOD activity, and it increased at 0.5 and 0.1 mM of CS-SA NCs compared to the control treatment. Also, the APX activity showed a significant increase in plants treated with salinity stress, so that the utmost activity was showed in 0.5 mM of CS-SA NCs (Fig. 3).

**Table 4:** Analysis of variance antioxidant Enzyme activity, H_2_O_2_ and total antioxidant of grapevine cv. ‘Sultana’ treated with CS-SA NCs under salinity stress.

S.O.V	df	H_2_O_2_	SOD	APX	GPX	CAT	Total antioxidant
Salinity	2	3719.451**	221.063**	61.373**	3.218**	0.005**	3.275**
Nanocomposite (NCs)	2	6721.831**	169.218**	21.331**	0.311^ns^	0.002**	1.996**
Salinity × NCs	4	343.762**	3.676*	2.065*	1.131**	0.000*	3.152**
Error	18	69.351	9.5	1.194	0.185	0.000	3.44
CV		8.97	4.51	23.54	13.97	9.03	5.47

*,**, ns significant at *p* ≤ 0.05 and *p* ≤ 0.01 respectively no significant.

### Nutrients content

**Figure 4:**
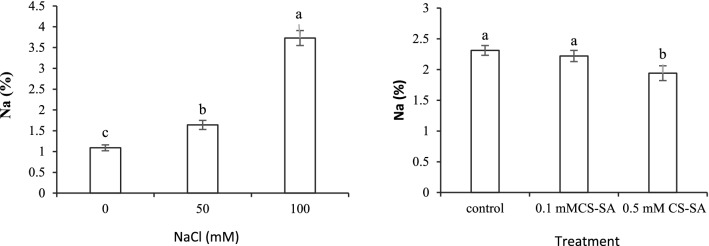
Effect of CS-SA NCs on Na of grapevine cv. ‘Sultana’. Means followed by the same letter on columns are not significantly different at 0.05 level, according to Duncan's multiple range test. Data are mean ± SD (n = 3 replicates).

Based to the results, N, P, K, Mg, Zn, Fe content, and Na^+^/K^+^ were significantly modified by salinity stress and foliar application of CS-SA NCs at 1% and 5% probability (Table 5). According to the findings, the N content declined significantly with increasing salinity levels, while CS-SA NCs foliar application ameliorated it at the different salinity levels. The P content lessedd significantly with increasing salinity stress, and the maximum content belonged to 0.5 mM of the CS-SA NCs treatment without salinity; the 100 mM NaCl without foliar application contained the lowest P level. The results revealed that the K content was minimal in 100 mM salinity treatment without CS-SA NCs foliar application, and the highest K level was obtained at 0.5 mM of CS-SA NCs without NaCl treatment. The lowermost of Mg content was recordedin the 100 mM salinity treatment without foliar application, which reduced with enhancing the salinity concentration. With increasing salinity stress, the Fe content showed a significant reduction in the lowest level in 100 mM of NaCl stress. The Zn content decreased significantly in the grape plants exposed to salinity stress. The application of CS-SA NCs at 0.5 mM could effectively improve the Zn content of the grape leaves under salinity stress. At different levels of salinity stress, the Na^+^/K^+^ ratio declined significantly in the plants treated with the CS-SA NCs foliar application concentrations (Table 5). The salinity- CS-SA NCs interaction was not statistically significant on the Na^+^ content. The data showed that the Na^+^ content increased significantly with increasing salinity levels compared to the control treatment. Na^+^ content showed a significant decrease in 0.5 mM of CS-SA NCs compared to the control treatment (Fig. 4).

**Table 5:** Mean comparisons for the effects of CS-SA NCs under salinity on the nutrient element content of grapevine cv. ‘Sultana’.

NaCl (mM)	CS-SA NCs (mM)	N (%)	P (%)	K (%)	Mg (%)	Zn (ppm)	Fe (ppm)	Na^+^/K^+^
	0	2.41 ± 0.02c	0.63 ± 0.01b	2.4 ± 0.03b	1.47 ± 0.02d	39.96 ± 3.17b	233.63 ± 3.18b	0.44 ± 0.01ef
0	0.1	2.57 ± 0.01b	0.66 ± 0.01b	2.52 ± 0.04b	1.62 ± 0.01b	41.83 ± 3.23b	239.9 ± 2.65ab	0.46 ± 0.02ef
	0.5	2.71 ± 0.02a	0.78 ± 0.02a	2.75 ± 0.04a	1.77 ± 0.03a	49.73 ± 2.9a	244.83 ± 3.17a	0.38 ± 0.02f
	0	1.39 ± 0.01f	0.33 ± 0.01d	1.33 ± 0.05e	1.41 ± 0.01e	24.5 ± 1.8de	128.3 ± 3.13d	1.32 ± 0.05d
50	0.1	1.52 ± 0.02e	0.38 ± 0.03d	1.63 ± 0.04d	1.47 ± 0.02d	30.96 ± 2.59cd	141.2 ± 2.13c	1.11 ± 0.07d
	0.5	1.79 ± 0.02d	0.53 ± 0.04c	2.03 ± 0.05c	1.54 ± 0.02c	38.6 ± 1.47bc	148.4 ± 2.98c	0.67 ± 0.02e
	0	1.18 ± 0.01h	0.19 ± 0.01f	1.02 ± 0.05g	1.15 ± 0.01g	14.86 ± 1.17f	121.53 ± 2.1d	3.89 ± 0.15a
100	0.1	1.27 ± 0.01g	0.26 ± 0.02e	1.15 ± 0.02fg	1.24 ± 0.02f	17.56 ± 0.61ef	126.5 ± 3.71d	3.24 ± 0.05b
	0.5	1.39 ± 0.01f	0.33 ± 0.02d	1.2 ± 0.03ef	1.29 ± 0.02f	21.43 ± 1.16ef	128.4 ± 1.82d	2.89 ± 0.06c
Significance								
Salinity		**	**	**	**	**	**	**
Nanocomposite (NCs)		**	**	**	**	ns	*	**
Salinity × NCs		**	**	**	**	**	*	**
Error		0.002	0.001	0.008	0.001	22.1	35.99	0.02
CV		2.55	7.87	5.09	2.63	15.14	3.57	8.9

Similar letters show no meaningful difference at 5% probability level by Duncan’s Multiple Range Test. Data are mean ± SD (n = 3 replicates). ns, ** and *: Non-significant, significant at 1 and 5 percentage probability levels, respectively.

### Multivariate analysis of *Vitis vinifera* cv. ‘Sultana’ under normal and salinity stress and CS-SA NCs treatments

**Figure 5:**
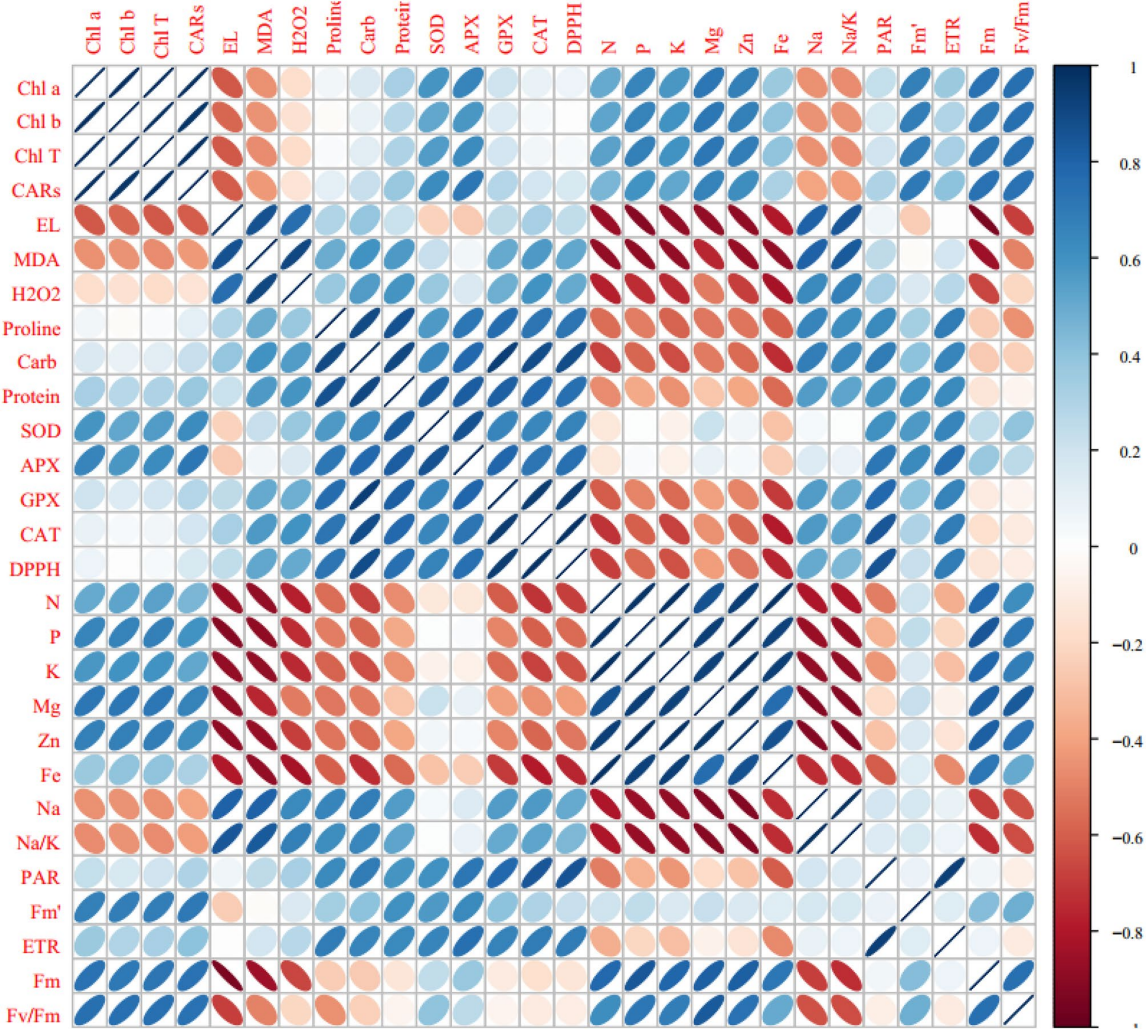
Heat map of Pearson’s correlation analysis for the response of *Vitis Vinifera* cv. ‘Sultana’ under salinity stress with application CS-SA NCs. Heat map representing of Chlorophyll *a* (Chl *a*), Chlorophyll *b* (Chl *b*), Total chlorophyll (Total Chl), Carotenoids (CARs), Electrolyte leakage (EL), Malondialdehyde (MDA), H_2_O_2_ content_,_ Proline content, Carbohydrate (Carb), Total soluble protein content, Superoxide dismutase (SOD) activity, Ascorbate peroxidase (APX) activity, Guaiacol peroxidase (GPX) activity, catalase (CAT), total antioxidant (DPPH), nitrogen (N), phosphorus (P), potassium (K), magnesium (Mg), zinc (Zn), iron (Fe), sodium (Na), Na/K, (PAR), (Fm'), electron transport rate (ETR), maximal fluorescence (Fm), maximum photochemical quantum yield of photosystem II (Fv/Fm).

Pearson’s correlations of chlorophyll fluorescence, photosynthetic pigments, antioxidant enzymes, and some biochemical traits are exhibited in Fig. 5. The findings showed that photosynthetic pigments, SOD, APX, N, P, K, Mg, Fm, Fm`, Fv/Fm positively correlated with each other, while significant negatively correlated EL, MDA, Na, Na^+^/K^+^, and H_2_O_2_. A negative significant correlation was recorded among EL, MDA, H_2_O_2_, proline, total soluble protein, and total soluble carbohydrate with N, P, K, and Mg content, but these traits positively correlated with Na, Na/k, APX, SOD, CAT, GPX, DPPH, Fm', ETR and PAR. A negative correlation was observed between Na and Na/K and other evaluated nutrients.

**Figure 6:**
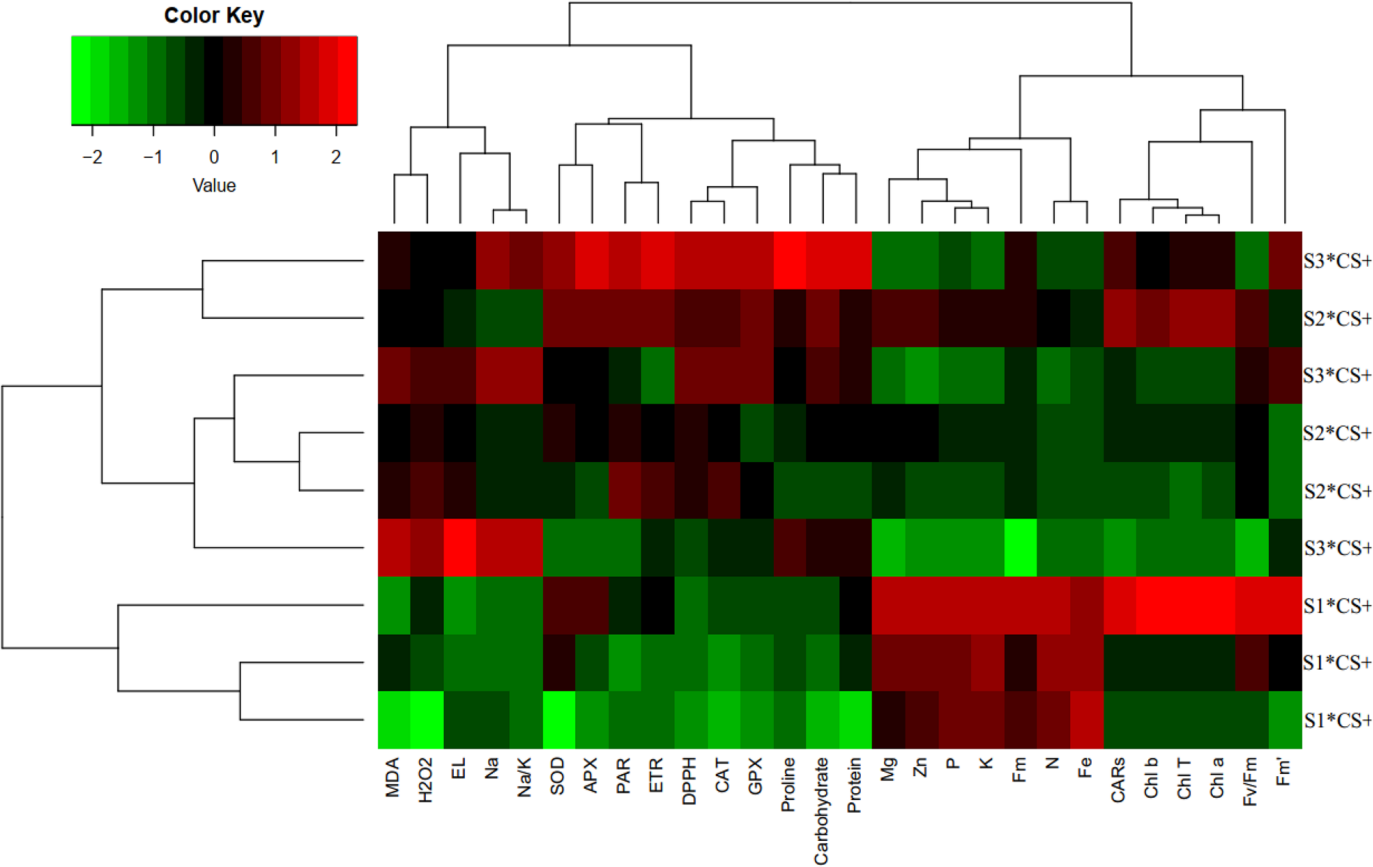
Heat map (a), loading biplot of the evaluated traits (b) and Principal component analysis heat map (C) of the enzymatic antioxidants pool, the biochemical changes, chlorophyll fluorescence and nutrient elements content in *Vitis Vinifera* cv. ‘Sultana’ under salinity stress with application CS-SA NCs. Heat map representing of Chlorophyll *a* (Chl *a*), Chlorophyll *b* (Chl *b*), Total chlorophyll (Total Chl), Carotenoids (CARs), Electrolyte leakage (EL), Malondialdehyde (MDA), H_2_O_2_ content_,_ Proline content, Carbohydrate (Carb), Total soluble protein content, Superoxide dismutase (SOD) activity, Ascorbate peroxidase (APX) activity, Guaiacol peroxidase (GPX) activity, catalase (CAT), total antioxidant (DPPH), nitrogen (N), phosphorus (P), potassium (K), magnesium (Mg), zinc (Zn), iron (Fe), sodium (Na), Na/K, (PAR), (Fm'), electron transport rate (ETR), maximal fluorescence (Fm), maximum photochemical quantum yield of photosystem II (Fv/Fm).

Heat map analysis based on the response of the plants to salinity and CS-SA NCs foliar application revealed that the traits including MDA, H_2_O_2_, EL, Na, Na/K, SOD, APX, PAR, ETR, DPPH, CAT, GPX, proline, total soluble protein, and total soluble carbohydrate had positive accordance to salinity stress, and on the other hand, the evaluated nutrient content, photosynthesis pigment, and fluorescence chlorophyll decreased with increasing salinity stress. The heat map analysis showed CS-SA NCs foliar application recovered the adverse effect of salinity stress by improving the physiological and nutritional traits (Fig. 6).

**Figure 7:**
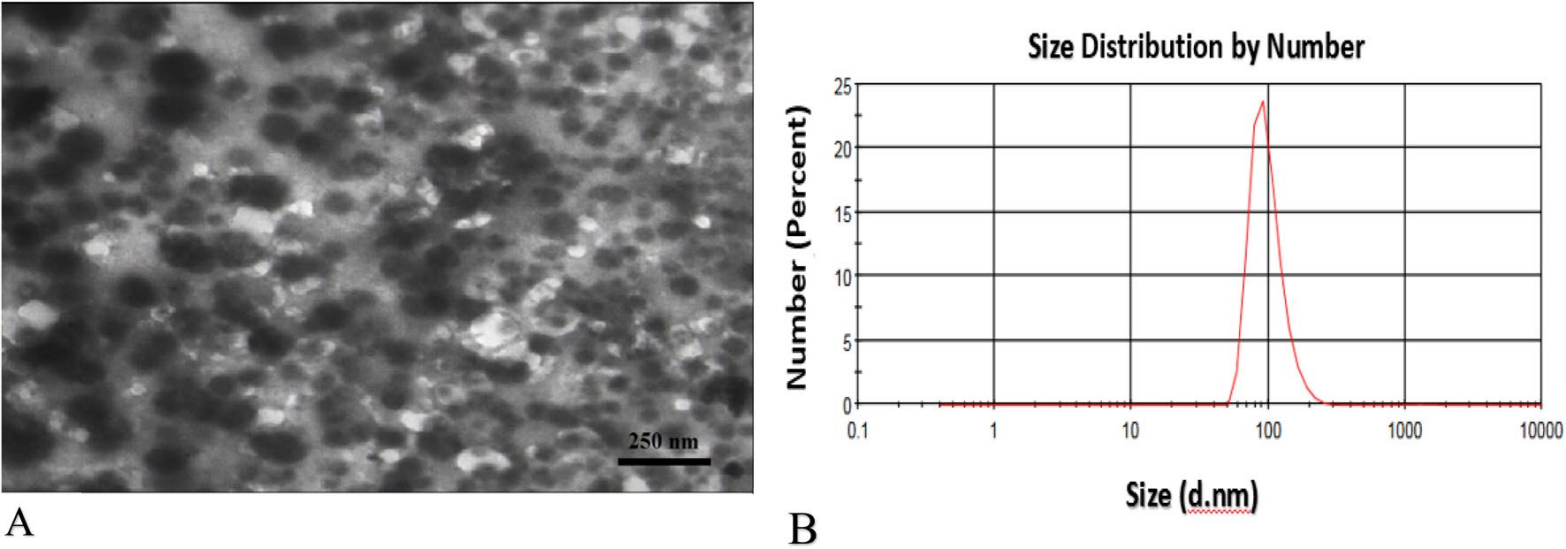
TEM image of sonochemical synthesis of CS-SA nanocomposite (**A**), and DLS analysis of CS-SA nanoparticles (**B**).

Cluster analysis and dendrograms in the heat map (Fig. 6) showed three main clusters in the evaluated features of the plants under salinity stress and CS-SA NCs foliar application. Cluster I comprised MDA, H_2_O_2_, EL, Na, Na/K, SOD, APX, PAR, ETR, DPPH, CAT, GPX, proline, total soluble protein and total soluble carbohydrate; cluster II comprised nutrients content and Fm; cluster Ш included photosynthesis pigments, Fm' and Fv/Fm (Fig. 7). In general, cluster analysis of heat maps for salinity stress combined with CS-SA NCs indicated three classes. Class I contained the plants under 50 and 100 mM of NaCl with 0.5 mM foliar application of CS-SA NCs; Class II contained the plants treated with 50 and 100 mM of NaCl with 0.1 mM Cs-SA NCs foliar application, as well as the raised plants under 50 and 100 mM of NaCl with no-foliar application. Finally, class III included the grape plants under normal conditions which were sprayed by 0.1 and 0.5 mM CS-SA NCs and also the control plants (Fig. 6).

## Discussion

The measurement of chlorophyll fluorescence is one of the important, simple, and non-destructive methods to evaluate photosynthetic efficiency. Plant response to salinity depends on the ability of PSII to respond to salinity stress^29^. Salinity reduces the quantum yield of the PSII electron transfer, the amount of light energy reaching the reaction center, and the complex involvement of oxygen. When plastoquinone (PQ) is oxidized under natural conditions, the electron transfer system has a minimum value of Fo. In salinity stress, however, Fo increases due to changes in the structure of the thylakoid membrane and damage to the PSII reaction centers^30,31^. The Fv/Fm index indicates the initial yield of photosystem II and acts as a stress indicator as it is sensitive to early plant responses to stress^32^. A decrease in the Fv/Fm index was reported in wheat under salinity stress^33^. Under stress conditions, reductions in Fm, Fv, and Fv/Fm can inhibit electron transfer from the PSII reaction center to electron transfer^34^. In sweet pepper, salinity stress influenced chlorophyll fluorescence parameters and caused a significant reduction in the maximum PSII yield (Fv/Fm). The useful role of chitosan was reported in increasing the production of protective metabolites, increasing the contents of N and K as well as the number of chloroplasts under stress, thereby improving the chlorophyll fluorescence parameter^35^. In a study of salinity stress on strawberry^32^ and sweet pepper^35^ induction of chlorophyll fluorescence of leaves increased with increasing salinity levels. Also, NPQ and F_0_ increased with increasing stress, but Fv/Fm and Fm decreased, which was consistent with our findings.

Based on the present results, a decrease in chlorophyll content was observed under salinity stress. Reductions of photosynthetic pigments under salinity stress may be caused by the deficiency in the leaf area responsible for light absorption and photosynthesis or may be due to chlorophyll degradation by increasing the activity of chlorophyll-degrading enzymes under salinity stress^36,37^. Other reasons for the reduction of photosynthetic pigments in salinity conditions include various types of ROS that cause chlorophyll degradation and damage to photosynthetic pigments^8^. According to our results, an increase in carotenoids with SA application was reported in tomato^38^, strawberry^39^, and myrtle (*Catharanthus*)^40^. Chitosan foliar application reduced the adverse effect of salinity stress by increasing the chlorophyll content. This increase was attributed to improvements in stomatal conductance, transpiration rate, and cell size and number^41,42^. Chitosan improved leaf chlorophyll content due to a higher nitrogen uptake, its transfer to leaves, and thus increasing chlorophyll pigments^42^. An increase in leaf chlorophyll content with the use of chitosan was reported in tomatoes^43^ and cucumber^44^. Our results in chlorophyll content under stress and chitosan treatment were similar to the results of other researchers^43–46^.

Proline protects cells by improving osmotic regulation, inhibiting ROS increase, and protecting the membrane structure^47^. Proline plays an essential role as an osmotic stabilizer as well as a stabilizer and protector of enzymes, proteins, and membranes^48^. An increase in proline content is an indicator of stress reduction^47^. In a study on salinity stress in tomatoes, proline content increased due to its role in the regulation and inhibition of ROS^43^. Similar to the results of our research, an increase in proline content was reported in chitosan-treated tomatoes^43^ and sunflower^47^ under salinity stress conditions. In this study, an increase in proline was observed in salicylic acid and chitosan treatments, which was consistent with the results of other researchers^43,47,49^. An increase in lipid degradation rate and MDA production was observed under stress conditions with the formation of ROS, leading to cell damage and destruction^43^, which corresponds to the present results. SA application in salinity stress reduced the amount of MDA in tomatoes^50^. Chitosan pretreatment under salinity stress increased the activity of antioxidant enzymes and reduced MDA levels and the negative effects of salinity stress^43,51^. SA application in stress conditions leads to the expression of genes in plants that produce proteins that activate signaling pathways and, ultimately, programmed cell death^52^. SA is reported to stimulate the synthesis of stress-related proteins by increasing nitrate reductase activity^53^ and increasing the content of abscisic acid^54^. Chitosan treatment also increased the content of total soluble proteins due to its role in increasing the expression of enzymes involved in glycolysis^55,56^. The increase in the production of malondialdehyde and decrease in degradation of lipids with the application of nano-chitosan-salicylic acid in the present results were consistent with the findings of other researchers^43,50^.

The activity of antioxidant enzymes increases in plants exposed to salinity stress because some antioxidant enzymes should be active to maintain lower levels of ROS^43,57^. SA activates the antioxidant enzymes SOD and CAT in stressed plants^13^. In myrtle, SA treatment in salinity stress increased the activity of SOD^58^. Chitosan can activate ROS-inhibitory systems in plants^43^. The use of chitosan as a bioelicitor with the potential to inhibit ROS has been shown in numerous studies. The activity of antioxidant enzymes increased significantly under the effect of chitosan treatment^59^. Chitosan could reportedly increase the activity of SOD and other antioxidant enzymes and cause tissue protection and delayed aging in stressed grapes^60^. Based on the results of the present study, the activity of superoxide dismutase and ascorbate peroxidase enzymes has increased significantly with increasing salinity and the application of salicylic acid-chitosan nanocomposite, which was consistent with the findings of other researchers in apple^61^, tomato^43^. The guaiacol peroxidase (GPX) enzyme is active in the cytosol, and glutathione is used as its cofactor^55^. This enzyme is present in the cellular and apoplasmic systems and is involved in many growth and development processes in the plant. Increasing salinity levels increased the activity of GPX^13^. The use of chitosan increased the activities of SOD, POX, and CAT in wheat and maize seedlings under salinity stress^62^. In the study of salt stress in spinach^63^, the activity of two enzymes, ascorbate peroxidase, and guaiacol peroxidase, increased significantly, which was consistent with our findings in grapes under salt stress.

NaCl salinity stress reduced the concentrations of Ca and Mg in all plant organs^64^. K uptake in salinity stress decreased significantly due to the effect of sodium on K transport in xylems and the inhibition of uptake processes^4^. Increased concentrations of Na^+^ and Cl^−^ because of salinity stress reduced the uptake of K^+^, Ca^2+^, and NO3^−^ and nutrient imbalance or deficiency^65^. In salinity conditions, a decrease in the Na^+^/K^+^ ratio in the plant indicates its resistance to salinity stress^9,64^. SA affects the intracellular ion balance of Na^+^ and K^+^ by increasing the regulation of H^+^-ATPase activity and thereby increases plant salinity resistance^66^, which is in agreement with our results. Decreased uptake of K and Ca has been reported under high salinity levels. Osmotic damage in plants occurs due to high levels of Na in leaf apoplasts^7,57^. SA significantly increased Fe uptake in strawberries^39^ and cucumber^67^. SA increases the amount of cytoplasmic K compared to Na by increasing the activity of the H^+^-ATPase pump in the cell membrane and providing a proton gradient, which contributes to reducing the toxic effects of Na and Cl and stimulates the activity of plant antioxidant systems and removal of ROS, thereby maintaining the integrity and protection of the cell membrane^68^. In the strawberry plant, leaf K content increased significantly by the effect of chitosan treatment^69^. Chitosan application in chickpea plants increased K content under salinity stress^70^. It seems that chitosan application caused the proper response of stressed grape plants to salinity stress by increasing and decreasing the concentrations of K and Na, respectively; in other words, chitosan could minimize ionic toxicity caused by salinity stress. In a study, the concentration of magnesium, calcium, potassium, iron and zinc elements decreased in the salinity stress of Selva strawberry^71^, which was consistent with our findings in grapes under salinity stress. At high levels of salinity, the ratio of sodium to potassium caused an ion imbalance, which is similar to the results of the present study, that salinity increased the ratio of sodium to potassium in cucumber plants^72^. In this study, salicylic acid treatment has reduced sodium ion concentration, which is in agreement with the findings of Jayakannan et al.^73^ in Arabidopsis.

## Methods

### Plant material and treatments

The current research was carried out in 2021 in the research greenhouse of the Department of Horticultural Science and Engineering, Faculty of Agriculture, located at Maragheh University, with a geographic location of 46,16° N latitude and 22,37° E longitude. The homogeneous one-year-old rooted cuttings of *Vitis vinifera* L. cv. ‘Sultana’ was provided by the nursery of the Horticultural Science Department, University of Maragheh, Iran by the relevant institutional and national guidelines and legislations. They were cultured in 5 L pots containing soil with a loamy sand texture (Table 6). For the initial growth and the adaptation of grapevines to greenhouse conditions (16 and 8 h of light and darkness, 30:25 °C day and night temperature). During the growth period, necessary care such as irrigation and other operations was taken regularly. After the full growth of the leaves of the seedlings, the treatments were carried out. To investigate the effects of foliar application of chitosan-salicylic acid nanocomposites (CS-SA NCs) on the physiological and biochemical properties of ‘Sultana’ cultivar grape in salinity conditions, a factorial experiment was used in completely randomized design (CRD) with three replications. One month after the establishment of the plants, salt stress was applied for one month, and during the stress period, the root environment of the plants was completely washed with salt-free water once every five days to minimize the changes in EC and pH due to washing, and nanocomposite foliar spraying Chitosan-salicylic acid was applied in two stages (the first stage two weeks after salt stress and the second stage at the end of salt stress). Treatments included: salinity stress at three levels (0, 50, and 100 mM NaCl) and foliar application of CS-SA NCs at three levels (0, 0.1, and 0.5 mM).

**Table 6:** Physicochemical properties of the soil sample utilized in the present experiment.

Clay (%)	Silt (%)	Sand (%)	K (ava) ppm	P (ava) ppm	Total N %	Organic carbon %	T.N.V. %	pH	EC × 10^3^
25	26	49	610	17.65	0.068	0.9	5.25	7.5	1.96

### Synthesis of chitosan-salicylic acid nanocomposite (CS-SA NCs)

To prepare CS-SA NCs, a biopolymer was used to load salicylic acid. In this study, 0.1 g of low molecular weight nanocomposite powder (25 ml of 1 wt% acetic acid solution was added and stirred for 2 h at 70 °C using a magnetic stirrer to obtain a clear CS solution. 100 μl SA of the prepared solution was added to the CS solution, then stirred rapidly for 1 h Sodium tripolyphosphate (TPP) was used as a cross linker with a ratio of 2.5 to CS content. TPP was dissolved in 5 ml of distilled water and then slowly added to the CS-SA solution, then rinsed several times with distilled water to remove the reaction material from the supernatant.

### Chitosan-salicylic acid nanocomposite (CS-SA NCs) characterization

Figure 7a represents the TEM image of fabricated CS-SA NCs. As shown, the successful octahedron nanoparticles are around 70–100 nm, which agrees with the DLS data presented in Fig. 7b.

### Chlorophyll Fluorescence Indices

Chlorophyll fluorescence was measured by a fluorometer (model: PAM 2500-WALZ, Germany) from the last fifth of leaves in the light. Minimal fluorescence (F0), photochemical quantum yield of photosystem II (Y(II)), electron transport rate (ETR), maximal fluorescence (Fm), Variable fluorescence (Fv), and maximum photochemical quantum yield of photosystem II (Fv/Fm) were assayed.

### Chlorophyll and carotenoid content

0.5 g of the sample was digested with 5 cc of 80% acetone and centrifuged for 10 min at 6000 rpm and read at 663, 645, and 470 nm. Chlorophyll content was determined according to Dere et al.^74^.

$${\text{Ca }}\left( {{\text{mg}}/{\text{g}}} \right) \, = \, \left[ {{12}.{7} \times {\text{A663 }}{-}{ 2}.{69} \times {\text{A645}}} \right] \, \times {\text{V}}/{1}000 \times {\text{W }}\left( {\text{Chlorophyll a}} \right)$$

$${\text{Cb }}\left( {{\text{mg}}/{\text{g}}} \right) \, = \, \left[ {{22}.{9} \times {\text{A645 }}{-}{ 4}.{86} \times {\text{A663}}} \right] \, \times {\text{V}}/{1}000 \times {\text{W }}\left( {\text{Chlorophyll b}} \right)$$

$${\text{Ca}} + {\text{b }}\left( {{\text{mg}}/{\text{g}}} \right) \, = \, \left[ {{8}.0{2} \times {\text{A663 }} + { 2}0.{2}0 \times {\text{A645}}} \right] \, \times {\text{V}}/{1}000 \times {\text{W }}\left( {{\text{Chlorophyll a}} + {\text{b}}} \right)$$

where V = volume of the extract (ml); W = Weight of fresh leaves (g).

### Proline content

Using Bates et al.^75^ method, 0.5 g of plant sample was first digested with 10 ml of 3% sulfosalicylic acid and after centrifugation, 2 ml of the extract, 2 ml of ninhydrin acid, and 2 ml of glacial acetic acid were mixed and placed in a bain-marie. Then, 4 ml of toluene was added, and it was read at 520 nm.

### Malondialdehyde

0.2 g of the plant sample was homogenized in 2 ml of 20% Trichloroacetic acid containing 0.05% TBA. The samples later were incubated atat 95 °C for 30 min and transferred to the ice. The samples were then centrifuged at 10,000 rpm for 10 min and the absorbance was measured at 532 and 600 nm. The extent of lipid peroxidation was obtained from the difference between the absorption wavelengths in the darkness coefficient of 155 mmol cm^−1^^76^.

### Electrolyte leakage content

Ion leakage was measured according to the Nayyar method^77^. The electrical conductivity of the samples was measured by reaching the ambient temperature as the initial EC by EC model CC-501. To measure the secondary EC, the samples were placed at 100 °C for 10 min. After reaching the EC ambient temperature again, the samples were measured as EC2 by EC meter, and finally the percentage of ion leakage from the product. The division of the primary EC into the secondary EC was calculated.

### Carbohydrate soluble content

To measure soluble carbohydrates, 3 ml of the alcoholic extract obtained was mixed with 1 ml of freshly prepared anthrone (330 mg of anthrone and 300 ml of 15% sulfuric acid). After cooling, the adsorption of the samples with the device the spectrophotometer (UV-1800 Shimadzu, Japan) was read at a wavelength of 653 nm^78^.

### Total soluble protein content

Reaction solution contained 100 μl of enzyme solution, 200 μl of Bradford reagent, and 700 μl of deionized water. 2 min after the complex formation; Bradford regent shows the highest integration with the amino acids. Absorbance was evaluated at 535 nm. The protein content of the samples was calculated based on a standard curve obtained from the defined amounts of bovine serum albumin^79^.

### Hydrogen peroxide

0.2 g of the plant material was homogenized in 2 ml of 0.1% Trichloroacetic acid and centrifuged at 12,000 g for 15 min. 0.5 ml supernatant was added to 0.5 ml of phosphate buffer (10 mmol, pH = 7) and 1 ml of Iodide potassium (1 mol). The sample's absorbance was measured at 390 nm. Standard curves were established with the different concentrations of Hydrogen peroxide^80^.

### Total antioxidant capacity

The antioxidant capacity of the extracts was calculated as the inhibition percentage of DPPH using the method of Chiou et al.^81^. 0.2 g of leaf tissue was digested with 2 ml of 80% methanol, then the resulting extract was centrifuged for 30 min at 4 °C and 5000 rpm. 100 μl of leaf extract was mixed with 1900 μl of DPPH solution and homogenized. Absorbance was evaluated at 520 nm.

### Antioxidant enzymes assay

For the extraction of Guaiacol peroxidase (GPX) and soluble proteins, 0.2 g of the sample was homogenized in liquid nitrogen. 2 ml phosphate buffer (pH = 7.5) containing EDTA (0.5 mol) was added. The samples were incubated at 4 °C for 15 min and were centrifuged at 15 rpm. Due to the instability and very low half-life of ascorbate peroxidase with ex-vivo conditions and for the keeping structure of the compound; we tried to use polyvinylpyrrolidone 5% and ascorbate (2 ml) to the respected enzyme solution.

### Guaiacol peroxidase (GPX) activity

For GPX activity, the reaction mixture was containing 1 ml phosphate buffer (100 mmol, pH = 7) along with EDTA (0.1 mmol), 1 mL guaiacol (15 mmol), 1 ml H_2_O_2_ (3 mmol), and 50 μL of the extracted enzyme solution. The reaction response was measured at 470 nm for 1 min. Enzymatic activity, based on the amount of tetraguaiacol, was obtained using a darkness coefficient of 26/6 m cm^−1^^82^.

### Ascorbate peroxidase (APX) activity

APX was assayed as; the reaction mixture was containing 250 μL phosphate buffer (pH = 7) along with EDTA, 10 μL H_2_O_2_ (1 mmol), 250 μL sodium ascorbate (0.25 mmol) and 50 μL enzyme solution. The absorbance was measured at 290 nm for 1 min. Enzymatic activity was calculated using the darkness coefficient of 2.8 mmol^−1^ cm^−1^. The resulting number indicates the activity of Ascorbate Peroxidase based on micromoles of oxidized Ascorbate per minute^82^.

### Superoxide dismutase (SOD) activity

SOD activity was determined by measuring the inhibition of light reduction of nitroblue tetrazolium at a wavelength of 560 nm. Doing this, 50 ml of 50 mM potassium phosphate buffer, pH: 7.5, was used. Then, 75 μM nitroblue tetrazolium, 13 μM methionine, 0.1 μM EDTA solution, and 4 μM riboflavin were added to the buffer and the solution was stored in a dark place^83^.

### Catalase (CAT) activity

0.5 g of grape leaf samples were homogenized with 0.1 M cold potassium phosphate buffer (pH: 7.5) with 0.5 mM EDTA based on the method of Dezar et al.^84^. From the resulting supernatant, 0.05 ml was added to 1.5 ml of 0.1 mM phosphate buffer (pH: 7) and 1.45 ml of double distilled water. The reaction was started by adding 0.5 ml of 75 mM hydrogen peroxide and a decrease in adsorption was recorded at 240 nm for 1 min.

### Leaves nutrient elements content

The flame photometric method (Corning, 410, England) was employed to measure the amount of sodium and potassium. The atomic absorption spectrometer (Corning, 410, England) was used to measure Zn, Fe, Ca, P, Mg, and Mn content (Corning, 410, England) and N content was quantified by Kjeldahl methods^85,86^.

The present experiment was performed as a factorial based on a completely randomized design with three replications. MSTATC (ver. 2.1, Michigan University), Minitab (ver. 17), and R software (ver. 3.6.3) were used for the ANOVA, cluster, biplot, and correlation analysis of data, respectively and, Excel (2016) was used to draw the figures. The means were compared using Duncan’s multiple range tests at 5 and 1% probability levels.

## Conclusion

The results of this research demonstrated that photosynthetic pigments decreased in ‘Sultana’ cultivar grape plants with increasing salinity stress, but it increased the content of osmolytes and antioxidant enzymes. Salinity interrupted ionic homeostasis and reduced nutrients. The application of the CS-SA NCs in stress and non-stress conditions positively affected the improvement of the studied traits of grape plants, improved nutrient levels, and reduced the Na level. Consequently, this nanocomposite represents an innovative approach that can be successfully used in grape plants to improve the yield under salinity stress. However, further validation is needed to determine their effectiveness in other plant species.

## Data Availability

The datasets used and analyzed during the current study are available from the corresponding author on reasonable request.

## Abbreviations

CAT: Catalase activity
APX: Ascorbate peroxidase activity
GPX: Guaiacol peroxidase activity
SOD: Superoxide dismutase activity
EL: Electrolyte leakage
F0: Minimal fluorescence
MDA: Malondialdehyde
Protein: Total soluble protein
RWC: Relative Water Content
Chlb: Chlorophyll b
Totalchl: Total chlorophyll
Chla: Chlorophyll a
Fm: Maximal fluorescence
PAR: Photosynthetically active radiation
F0: Minimal fluorescence
ETR: Electron transport rate
Fv: Variable fluorescence
Fv/Fm: The ratio of variable fluorescence to maximal fluorescence
N: Nitrogen
P: Phosphor
K: Potassium
Mg: Magnesium
Zn: Zinc
Fe: Iron
